# Treatment of Cold Agglutinin Syndrome Secondary to Chronic Lymphocytic Leukemia With Sutimlimab and Obinutuzumab–Venetoclax

**DOI:** 10.1155/crh/9060134

**Published:** 2026-08-07

**Authors:** Coen J. Lap, Catherine M. Broome

**Affiliations:** ^1^ Division of Hematology, Lombardi Comprehensive Cancer Center, Medstar Georgetown University Hospital, Washington, DC 20007, USA, medstargeorgetown.org

**Keywords:** CAS, CLL, cold agglutinin syndrome, obinutuzumab, sutimlimab, venetoclax

## Abstract

Cold agglutinin–mediated autoimmune hemolytic anemia (AIHA) is a rare disorder in which IgM autoantibodies lead to complement‐dependent hemolysis and cold‐induced circulatory symptoms. It is categorized as either cold agglutinin disease (CAD), a primary lymphoproliferative disorder (LPD), or cold agglutinin syndrome (CAS), which occurs secondary to other conditions most commonly infections or lymphoid malignancies. Although the treatment for CAS should be directed toward the underlying condition, the often slower response rates of these treatments, especially in the setting of LPD, could necessitate other strategies for patients with severe hemolytic anemia requiring more rapid control of hemolysis. For patients with CAD, inhibition of the classical complement pathway with the C1s inhibitor sutimlimab has demonstrated significant control of hemolysis, resolution of anemia, and improvement in quality of life. However, little has been published regarding the use of sutimlimab for the treatment of CAS. Here, we describe a patient with CAS secondary to chronic lymphocytic leukemia (CLL) with severe IgM‐driven complement‐mediated hemolysis who was successfully treated with a combination of sutimlimab and CLL‐directed therapy with obinutuzumab–venetoclax. Sutimlimab provided rapid cessation of hemolysis, acting as a bridge while the obinutuzumab–venetoclax addressed the underlying CLL that was presumably responsible for the autoantibody production. This case demonstrates that combining sutimlimab with obinutuzumab–venetoclax is an effective and safe treatment for patients with CAS in the setting of CLL, supporting the use of short‐term sutimlimab for CAS as a bridge to more durable treatment for underlying LPD such as CLL.

## 1. Introduction

Cold agglutinin (CA)–mediated autoimmune hemolytic anemia (AIHA) accounts for 15%–25% of all AIHA and is characterized by complement‐dependent hemolysis due to the presence of autoantibodies termed CA [1, 2]. These autoantibodies, typically of the IgM subclass, transiently bind the “I” antigen on the membrane of erythrocytes when subjected to temperatures below core body, resulting in agglutination of erythrocytes and ultimately activation of the classical complement pathway and extravascular hemolysis. CA‐mediated AIHA can be divided into cold agglutinin disease (CAD) and cold agglutinin syndrome (CAS). CAD is a distinct clinicopathologic entity and considered a clonal lymphoproliferative disorder usually associated with monoclonal IgM. In contrast, CAS occurs secondary to infections, autoimmune disorders, or lymphoid malignancies. While infection‐ or autoimmune‐associated CAS frequently involves polyclonal antibodies, lymphoid malignancies also typically present with monoclonal IgM [3, 4]. Symptoms of CA‐mediated hemolysis can vary dramatically, from chronic, compensated hemolysis to life‐threatening hemolytic crises [5]. Additionally, erythrocyte aggregation can cause cold‐induced circulatory symptoms, including acrocyanosis [6]. Therapeutic approaches can be directed either the underlying clonal lymphoproliferation or the complement‐mediated hemolysis, whereas in CAS, treating the underlying disease generally resolves the CA‐mediated hemolysis [7]. However, immunochemotherapy for underlying lymphoid malignancies usually requires weeks to months of treatment to observe responses, necessitating other strategies for patients with severe CA‐mediated hemolytic anemia. Selective upstream inhibition of the classical complement pathway with the C1s inhibitor sutimlimab has been shown to rapidly halt complement‐mediated hemolysis in patients with CAD, though responses are short‐lived upon discontinuation as complement inhibition does not address the underlying autoantibody production [8, 9]. There are limited data using sutimlimab as a bridge therapy for CAS toward more definitive treatment of the underlying driver of autoantibody production. Here, we present a patient with CAS secondary to chronic lymphocytic leukemia (CLL) who was successfully treated with a combination of sutimlimab and obinutuzumab–venetoclax. This approach resulted in rapid cessation of complement‐mediated hemolysis allowing timely CLL‐directed treatment to achieve a more durable response.

## 2. Case Description

A 61‐year‐old female presented in 2018 with cold‐dependent acrocyanosis and livedo reticularis. She first noticed these symptoms 8 years prior, though no further workup had been pursued. Laboratory testing with temperature control revealed mild compensated hemolysis (Hgb 12.3 g/dL, MCV 109 fL, reticulocyte count 4.9%, haptoglobin < 10 mg/dL, bilirubin total 1.5 mg/dL and indirect 1.0 mg/dL, and lactate dehydrogenase 790 U/L) (Table 1). A normal platelet count (PLT 266 × 10^3^/μL) *and* white blood cell count (WBC 4.9 × 10^3^/μL) were observed with an absolute lymphocyte count of 2.9 × 10^3^/μL. Vitamin B12 level was 826 pg/mL and folate level > 20 ng/mL. A direct antiglobulin test (DAT) was strongly positive for C3d only, with the presence of high titer CA at 4°C (> 1:512). Hypocomplementemia was present with low C3 and C4 (C3 74 mg/dL; C4 < 2 mg/dL). Serum protein electrophoresis with immunofixation demonstrated an IgM kappa paraprotein quantified at 0.8 g/dL. IgM level was elevated at 808 mg/dL. Bone marrow evaluation revealed a slightly hypercellular marrow with approximately 10% involvement of a monoclonal population of small lymphocytic cells positive for CD5, CD20, CD23, and PAX5 with kappa‐restriction, and negative for CD10, CD38, CD138, and CD34, an immunophenotype consistent with CLL. Peripheral blood flow cytometry showed involvement with approximately 8% monoclonal CD5+ B cells with kappa‐restriction (0.23 × 10^3^/μL). Fluorescence in situ hybridization detected trisomy 12, and molecular analysis was negative for mutations in *TP53* and *MYD88*. CT imaging did not show lymphadenopathy but did reveal pulmonary embolism for which anticoagulation was initiated. A diagnosis of CAS in the setting of CLL‐type monoclonal B‐cell lymphocytosis (MBL) was made. Because of limited symptoms and patient preference, a “watch‐and‐wait” strategy was pursued.

**TABLE 1:** Pertinent laboratory findings.

	10/25/2018 diagnosis	10/15/2024 before treatment	11/7/2024 before treatment	12/3/2024 ∼2 weeks after start sutimlimab + acalabrutinib	12/12/2024 on sutimlimab ∼1 wk stop acalabrutinib	12/23/2024 on sutimlimab before start obinutuzumab	1/3/2025 on sutimlimab ∼1 wk after start obinutuzumab	2/25/2025 on sutimlimab ∼1 mo after start venetoclax	7/22/2025 ∼2 months after stopping sutimlimab	Reference range
Hemoglobin, g/dL	12.3	8.5	7.7	6.1	6.2	7.4	8.6	9.6	12.3	11.0–14.5
MCV, fL	109	101	112	111	111	107	99	86	97	81–100
PLT, per cm^3^	266	284	277	269	307	484	325	400	152	145–400
WBC, per cm^3^	4.9	6.9	6.1	5.8	4.8	7.3	5.6	3.15	5.3	4.0–10.8
ALC, per cm^3^	2.9	2.8	2.6	0.3	2.0		1.3	0.9	1.2	0.6–4.9
Reticulocyte count, %	4.9	7.5	10.9				5.9	3.1	3.1	0.5–2.0
Total bilirubin, mg/dL	1.5	3.2	2.7	2.5	2.5	2.0	1.3	1.4	1.5	0.2–1.1
LDH, U/L	790	1221	1712	1318			1295	805	519	120–246
Haptoglobin, mg/dL	< 10									30–200
IgM, mg/dL	808		2061		1920			1230	827	26–217
Direct antiglobulin test	C3d positive				C3d positive	C3d positive		C3d positive		Negative
Cold agglutinin titer	> 1:512		1:4096					> 1:512	> 1:512	< 1:32

*Note:* PLT, platelet count; WBC, white blood cell count; LDH, lactate dehydrogenase; IgM, immunoglobulin M; wk, week; mo, months.

Abbreviations: ALC, absolute lymphocyte count; MCV, mean corpuscular volume.

**FIGURE 1:**
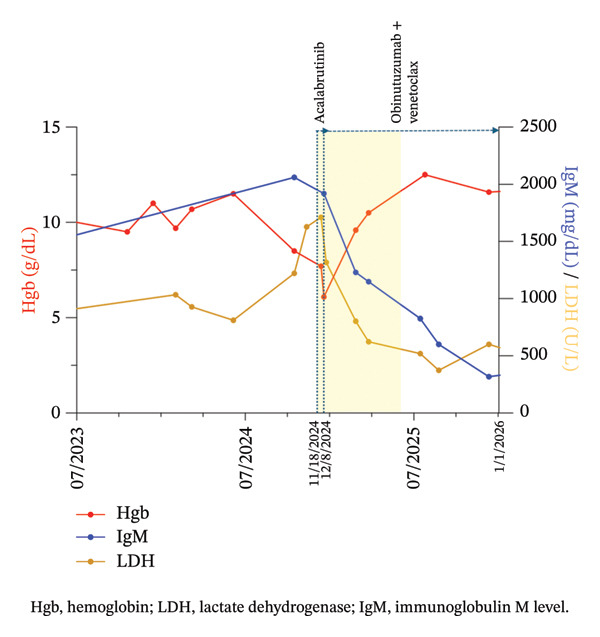
Trend of Hgb, LDH, and IgM in relation to treatment. The yellow shaded area indicates the time window during which the patient received sutimlimab.

She remained under surveillance but developed worsening fatigue and acrocyanosis in 2024. Repeat labs revealed worsening anemia (Hgb 7.7 g/dL; MCV 112 fL) and evidence of ongoing hemolysis (reticulocyte count 10.9%; total bilirubin 2.7 mg/dL; LDH 1,712 U/L; CA titer 1:4096; IgM 2,061 mg/dL) (Table 1). Absolute lymphocyte count fluctuated around 3.0 × 10^3^/μL. A repeat bone marrow evaluation revealed over 50% involvement with known CLL. Options for treatment of CLL were discussed. However, due to severe symptomatic anemia, rapid control of CAS‐mediated hemolysis was necessary for the patient to tolerate therapy for the underlying CLL (Figure 1). Sutimlimab was initiated in combination with CLL‐directed treatment with the Bruton tyrosine kinase (BTK) inhibitor acalabrutinib on November 18, 2024. Vaccinations against encapsulated bacteria were updated prior to the start of sutimlimab and daily antibiotic prophylaxis with azithromycin was provided. Within 2 weeks, worsening anemia developed with persistently elevated hemolysis parameters as well as debilitating weakness prompting discontinuation of acalabrutinib on December 3, 2024. After discontinuation of acalabrutinib, anemia started to improve and the weakness gradually resolved. At that time, the decision was made to continue sutimlimab and switch CLL‐directed treatment to obinutuzumab with venetoclax. Obinutuzumab was started on December 23, 2024. Venetoclax was initiated during cycle 2 of obinutuzumab and dose‐escalated in a stepwise fashion. Four weeks after starting sutimlimab, hemolysis parameters began to improve with concomitant rise in Hgb. Six months after the start of treatment, Hgb had normalized and fatigue and acrocyanosis symptoms had returned to baseline. She tolerated the treatment well, without signs of tumor lysis, infections, or hematologic toxicity. As of May 2025, treatment with obinutuzumab was completed and sutimlimab has been discontinued. Five months after discontinuation, her Hgb has remained within normal limits, and IgM level continues to trend down.

## 3. Discussion

Autoimmune phenomena may precede or accompany lymphoid malignancies, especially CLL [10]. AIHA accompanies CLL in approximately 5%–10% of patients and is often the result of warm IgG autoantibodies [11, 12]. However, in up to 10% of patients, IgM autoantibodies are identified. In contrast to polyclonal IgG autoantibodies, which are produced by nonmalignant self‐reactive B cells, cold IgM autoantibodies are produced by the malignant B cells and frequently have *κ* light‐chain restriction [13, 14]. The presence of trisomy 12 in CLL is associated with CAS and, similar to CAD, recurrent mutations in *CARD11* and *KMT2D* have been identified in CAS associated with CLL [15–17].

Pathologic CAs exist at titers of 1:64 or higher and generally possess a thermal amplitude over 28°C–30°C [18]. Although these autoantibodies remain unbound from erythrocytes at core body temperature, they bind to erythrocytes as blood circulates into the cooler peripheral circulation. Once bound, the antigen–antibody complex not only promotes hemagglutination but also binds to complement protein C1q, thereby initiating the generation of the C1 complex resulting in classical complement pathway activation. This ultimately results in C3b deposition on erythrocytes. C3b subsequently acts as a powerful opsonin causing predominantly extravascular hemolysis mediated by the monocyte–macrophage system in the liver. IgM autoantibodies can be polyclonal or monoclonal, each linked to different origins and prognosis [19]. Polyclonal IgM autoantibodies are often seen in the postinfectious setting, are generally less pathogenic, not due to lower titers, but because of a lower thermal amplitude, and frequently self‐resolve [2, 20]. Conversely, monoclonal IgM autoantibodies are typically seen in patients with an underlying lymphoproliferative disorder, have higher thermal amplitudes reacting at temperatures over 30°C–32°C, cause significant hemolysis, and rarely self‐resolve.

Treatment recommendations for CAS secondary to CLL are largely based on expert opinions and often extrapolated from treatment experience with CAD. Although rituximab therapy for CAD achieves partial responses in 50% of patients, median time to response is 1.5 months with a median duration of 6.5–11 months [21, 22]. Adding bendamustine or fludarabine to rituximab for the treatment of CAD can increase response and remission rates, but this comes with toxicities and long‐term risks [23, 24]. BTK inhibitors have shown efficacy in CAD and can induce relatively rapid responses, including in CAS secondary to CLL [25]. However, they do come with their own side effects and require indefinite treatment. More recently clinical trials for CAD have focused on complement inhibitors to halt complement‐mediated hemolysis. Although the C5 inhibitor eculizumab has been evaluated for CAD, responses have been disappointing likely because it does not inhibit the extravascular hemolysis mediated by classical pathway activation [26]. In contrast, the monoclonal antibody sutimlimab inhibits C1 complex formation by binding to C1s, thereby inhibiting classical pathway activation. Inhibition of C1s has been found to rapidly halt hemolysis and improve Hgb in patients with CAD in both the CADENZA and CARDINAL trials [8, 9].

Although the decision was made to start treatment with sutimlimab and acalabrutinib, worsening anemia and debilitating weakness attributed to the acalabrutinib prompted a change to an alternative first‐line CLL treatment, the CD20 monoclonal antibody obinutuzumab with the BCL2 inhibitor venetoclax [27–29]. While switching to zanubrutinib could have been considered due to its different side‐effect profile, we ultimately chose an agent with a different mechanism of action in the hope of achieving better tolerability. Two months after discontinuing the sutimlimab, Hgb remained within normal values. No major side effects were observed during treatment. This case highlights that sutimlimab combined with obinutuzumab–venetoclax can be a highly effective treatment for CAS in the setting of CLL. It also adds to the growing evidence that using short‐term sutimlimab for CAS as a bridge to more durable treatment for underlying disorders like CLL can be a highly effective and safe strategy. Treatment with sutimlimab for CAS can be considered regardless if the underlying disorder is treatment‐naïve or relapsed/refractory. The concept of utilizing sutimlimab as a bridge is further supported by a recent case report where sutimlimab provided a window for more definitive therapy in a patient with refractory CAS secondary to a plasma cell dyscrasia [30]. In both instances, rapid cessation of hemolysis allowed treatment to address the underlying process of IgM autoantibody production. While CAS can be an indolent and chronic disorder, it can also become severe due to more rapid hemolysis especially in those patients with underlying B‐cell malignancies. In those patients with CAS secondary to CLL, for whom rapid treatment is needed, a combination of sutimlimab and obinutuzumab–venetoclax could be considered.

## 4. Conclusion

**FIGURE 2:**
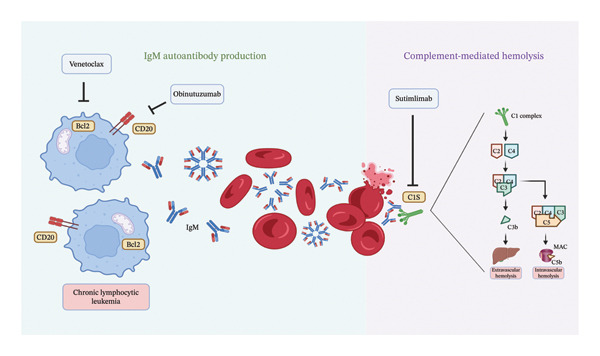
Treatment of cold agglutinin syndrome secondary to CLL with a combination of sutimlimab and obinutuzumab plus venetoclax. Inhibition of complement‐mediated hemolysis with sutimlimab provides a bridge toward a more durable response by treating the underlying chronic lymphocytic leukemia, thereby suppressing IgM autoantibody production.

For patients with CAS secondary to CLL requiring rapid control of hemolysis, using short‐term sutimlimab as a bridge toward more durable treatment for CLL with obinutuzumab–venetoclax is a highly effective and safe treatment strategy (Figure 2).

## Author Contributions

All authors contributed equally.

## Funding

The authors have nothing to report.

## Ethics Statement

The authors have nothing to report.

## Consent

Consent was obtained from the patient for publication of the manuscript.

## Conflicts of Interest

Catherine M. Broome has received research support and honoraria from Sanofi and Recordati. The other author declares no conflicts of interest.

## Data Availability

All figures and tables are originals and are available upon request.
